# Prevalence and correlates of erectile dysfunction among long-distance commercial vehicle drivers and commercial motorcycle riders in Ibadan Nigeria: a comparative cross-sectional study

**DOI:** 10.4314/ahs.v22i3.3

**Published:** 2022-09

**Authors:** Adekunle Adesola, Martins Imhasoloeva, Adesanmi Akinsulore

**Affiliations:** 1 Department of Psychiatry University College Hospital, Ibadan, Nigeria; 2 Department of Public Health Research Lumenplus Consulting, Ibadan, Nigeria; 3 Department of Mental Health Obafemi Awolowo University, Ile-Ife, Nigeria

**Keywords:** Erectile dysfunction, commercial motorcycle riders, long-distance commercial vehicle drivers, Southwest Nigeria

## Abstract

**Background:**

Erectile dysfunction (ED) is a global public health problem that affects the quality of life, interpersonal, occupational, and social functioning of sufferers. Despite being high-risk groups, there is a paucity of data on erectile dysfunction among commercial vehicle drivers and motorcycle riders.

**Objectives:**

We aimed to determine and compare the prevalence and factors associated with ED among long-distance commercial vehicle drivers (CVDs) and commercial motorcycle riders (CMRs) in Ibadan, Nigeria.

**Methods:**

We used a comparative cross-sectional study design to enroll eligible male respondents in selected motor/motorcycle parks within Ibadan metropolis. Interviewer administered questionnaires were used to elicit sociodemographic/health-related characteristics, and ED status among participants. Data was analyzed using STATA version 12. Chi-square and Binary logistic regression were conducted to explore the association between ED and other covariates. Analyses were performed at 5% significance level.

**Results:**

The prevalence of ED was significantly higher among motorcycle riders than vehicle drivers (71.4% vs 47.4%, p = 0.001). Predictors of ED among CMRs were monogamous marriage type and history of perineal injury; while among CVRs were aged above 40-years, history of perineal injury, and current use of alcohol.

**Conclusion:**

There is a need for public education and awareness programmes on ED to reduce the burden and improve well-being in these populations.

## Introduction

Erectile dysfunction (ED) is one of the most frequently self-reported sexual problems in men^1^ with significant impact on interpersonal relationships and quality of life of both the sufferer and the partner^2^. Globally, the prevalence of erectile dysfunction in the general population ranges from 15% to 66.9% across different countries of the world^2^–^10^, and it is projected that 322 million men would have erectile dysfunction by 2025^11^.

Several studies have reported on the correlates of erectile dysfunction in the general population. Findings from previous studies showed that medical conditions (hypertension, diabetes mellitus, obesity, heart disease, lower urinary tract symptoms), unhealthy lifestyles (smoking, use of alcohol, physical inactivity), use of antihypertensive drugs, history of perineal injury, previous perineal surgery especially prostate surgery and psychological factors (psychological stress, increased anxiety, depression, interpersonal problems, poor quality of life) were significantly associated with erectile dysfunction^2^,^3^,^5^,^7^,^9^–^12^. Of these factors, increasing age and diabetes mellitus had the strongest association^2^,^7^.

To the best of our knowledge, there is no empirical information on the prevalence and determinants of ED among long-distance commercial vehicle drivers and commercial motorcycle riders, nor is there any published data comparing the prevalence between these populations in our settings.

In previous studies conducted in Japan, the authors compared the association between erectile dysfunction and motorcycling among non-commercial male motorcyclists and healthy controls. These studies showed a significantly higher rate of ED among male motorcycle riders compared to the control group across all age groups^13^,^14^. Furthermore, ED was significantly predicted by increasing age, motorcycling and the presence of lower urinary symptoms particularly storage symptoms^13^,^14^. The mechanisms proposed for the relationship include the compression of the perineum by the saddle and the vibration of the engines during motorcycling which may affect the perineal neuro-vasculature^13^.

Long-distance commercial vehicle drivers and commercial motorcycle riders are at risk of erectile dysfunction due to the nature of their work. While the pressure and vibration to the perineum during motorcycling could increase the risk for ED among motorcycle riders, chronic back pain following long-distance journeys might increase the likelihood of erectile dysfunction among commercial drivers. In addition, most men who engage in these occupations often indulge in harmful behavioural and lifestyle practices such as alcohol and other drug abuse which are important risk factors for erectile dysfunction^15^,^16^. Also, the incidence of ED is gradually increasing particularly among men younger than 40 years who constitute the vast majority of commercial motorcycle riders and vehicle riders^17^.

Despite the abundance of risk factors for erectile dysfunction in these populations, empirical information on the prevalence and determinants of erectile dysfunction is scarce. To the knowledge of the authors, no previous study has assessed for the prevalence and correlates of ED among long-distance commercial vehicle drivers and commercial motorcycle riders, nor compared the prevalence of ED among them. Also, while there are group-specific risk factors such as trauma to the perineum in motorcycling and chronic back pain in long-distance journeys, the role played by each on ED is largely unknown. This study therefore aimed at determining and comparing the prevalence and factors associated with erectile dysfunction between long-distance commercial vehicle drivers and commercial motorcycle riders in Ibadan, Southwest Nigeria. This will help to determine the burden of erectile dysfunction among these high-risk groups and provide public education and awareness strategies and programmes that will help reduce the burden and impact of ED in these populations by adopting healthy lifestyles. Comparing these groups will help to determine difference in burden of ED and identify possible group-specific correlates which may inform interventions and policies for reducing the burden in each group.

## Materials And Methods

This was a comparative cross-sectional study carried out at selected major motor parks within Ibadan metropolis, the capital city of Oyo State from June 2019 to August 2019. Ibadan metropolis consists of about 12 major motor parks where intra-city and intercity motor vehicles convey passengers to different destinations within and outside the city. These motor parks are organized into unions, with each park headed by a chairman alongside other executive members. Similarly, motorcycle parks exist within the city and are clustered within the 11 local government areas (LGAs).

For this study, 6 LGAs were randomly selected for the recruitment of the study participants. They included Ibadan North, Ibadan Northwest, Ibadan Northeast, Ibadan Southwest, Oluyole and Akinyele LGAs. The target populations were long-distance commercial vehicle drivers at motor parks covering a distance of at least 400 kilometres daily and commercial motorcycle riders at motorcycle parks within the randomly selected LGAs. Eligibility criteria for this study included adult males (aged 18 years and above), sexually active and active engagement in commercial vehicle driving or motorcycle riding in the 6 months prior to the study.

### Sample size calculation

The sample size was determined using the formula for comparison between two groups.

Sample size (n) = 2(Za/2 + Zβ)^2^ P (1 − P)

(P1 – P2)^2^

Where

N = estimated sample size

Za/2 = 1.96 corresponding to standard normal deviate at a set at 5% level of significance

Zβ = 0.842 corresponding to 80% power

P = Pooled estimated prevalence from previous study proportion of variable of interest (P1 – P2)/2

P1= Prevalence of erectile dysfunction among men in the community estimated at 58.9% (3).

P2= Prevalence of erectile dysfunction among motorcycles riders estimated at 69.0% (8).

Therefore,

N = 2(1.96 + 0.842)^2^ * 0.934(1 – 0.934) = 196.4

(0.589 – 0.690)^2^

Assume a 10% non-response rate

N = 215 per group or 430 total sample size.

### Sampling method

A multistage cluster random sampling technique was used to recruit eligible long-distance commercial vehicle drivers and motorcycle riders. Ibadan has eleven LGAs from which 6 were randomly selected namely Ibadan North, Ibadan Northwest, Ibadan Northeast, Ibadan Southwest, Oluyole and Akinyele LGAs. For long-distance drivers, we identified 12 motor parks or clusters within the selected 6 LGAs and randomly selected 8 clusters. Subsequently, we sampled all eligible and consenting long-distance commercial vehicle drivers aiming 27 participants per cluster until the sample size was achieved.

Similarly, for commercial motorcycle riders, the motorcycle parks are often more dispersed and located at the foot of busy streets as well as market areas. We identified twenty motorcycle clusters within the six randomly selected LGAs from which we randomly selected 9 clusters. Thereafter, we sampled all eligible and consenting commercial motorcycle riders aiming for 24 participants per cluster until the sample size for the sub-group was achieved.

### Research Instruments

Socio-demographic and clinical questionnaire: this is a short questionnaire designed by the author to collect the socio-demographic and clinical data of eligible participants such as age, marital status, history of hypertension, diabetes etc.

International Index of Erectile Function (IIEF-5): this is an abridged, reliable and valid self-administered 5-item questionnaire used to measure erectile function. The score for the IIEF ranges from 5 to 25. Erectile function is classified into four classes based on these scores^18^; Severe ED 5–7, moderate ED 8–16, mild ED 17–21 and normal 22–25. Hence, the higher the score, the better the erectile function. The instrument has been used in Nigeria^2^,^8^,^19^–^21^ with a reliability coefficient (Cronbach's alpha) of 0.9201^21^.

### Data Collection Methods

Two research assistants were trained in the use and administration of the above-listed questionnaires. With the researcher, eligible participants were approached at the selected parks and written informed consent were obtained. The questionnaires were subsequently administered to the participants. The questionnaire was administered either in English or Yoruba language. The Yoruba version of the questionnaire was derived by standard iterative procedures of translation and back-translation. It was validated for this study with a reliability coefficient (Cronbach's alpha) of 0.8178.

### Data analysis and management

Data was entered into a Microsoft Excel spreadsheet and transferred into STATA software (StataCorp, College Station, version 12) for statistical analysis. Categorical variables were presented as frequency and percentages while continuous variables were summarized as mean and standard deviation. The dependent variable was erectile dysfunction (ED), which was based on the Index of Erectile Function (IIEF-5) scale. ED was dichotomized as either absence (participants with IIEF scores ranging between 22 to 25); or presence (participants with scores less than 22 corresponding to either mild, moderate, or severe score ranges on the IIEF scale) of ED. We performed both bivariate and multivariate analysis separately for each individual group. The association between ED and other sociodemographic/health related variables were explored using Chi-square tests. Variables that were statistically significant at bivariate analysis were included in a binary logistic regression model to determine the independent predictors of ED among each of the groups. All analyses were two-tailed with level of significance set at p<0.05.

### Ethical considerations

Ethical approval was obtained from the ethical review committee of the Oyo State Ministry of Health while permission was sought from the National Union of Road Transport Workers (NURTW), Ibadan branch and the Amalgamated Commercial Motorcycle Owners and Riders Association of Nigeria (ACOMORAN), Ibadan branch before the commencement of the study. The study was conducted according to the Declaration of Helsinki. Participants' confidentiality was maintained by anonymizing and coding the questionnaires using serial numbers. Participation in this study was entirely voluntary, and participants could decline or withdraw from the study without any consequence. Participants with erectile dysfunction were counselled to present for specialist assessment and management at the state hospital.

## Results

Eligible participants who gave informed consent and completed the survey were 204 commercial motorcycle riders and 219 long-distance commercial vehicle drivers, accounting for 98.4% and 100% response rates respectively. The long-distance drivers were significantly older than motorcycle riders (42.2 vs 34.6, p = 0.001). Compared to motorcycle riders, long-distance drivers were more likely to have over 10 years of driving experience (χ2 = 97.41, p = 0.001), make over 3500 Naira ($10) as average daily income (χ2 = 83.50, p = 0.001), ever used alcohol (χ2 = 12.83, p = 0.001) and ever smoked cigarette (χ2 = 5.97, p = 0.015). Table 1

**Table 1:** Socio-demographic and health-related characteristics of commercial motorcycle riders and long-distance commercial vehicle drivers

	Motorcycle Riders (n=204)	Vehicle Drivers (n=219)	χ^2^	p-value
**Age (years)**				
< 40	151 (74.02)	93 (42.47)	43.080	0.001*
≥ 40	53 (25.98)	126 (57.53)		
**Religion**				
Islam	98 (48.04)	127 (57.99)	4.201	0.040*
Christianity	106 (51.96)	92 (42.01)		
**Educational status**				
Primary	52 (25.49)	75 (34.25)	13.334	0.001*
Secondary	117 (57.35)	130 (59.36)		
Tertiary	35 (17.16)	14 (6.39)		
**Marital status**				
Not currently married	48 (23.53)	19 (8.68)	17.481	0.001*
Currently married	156 (76.47)	200 (91.32)		
**Type of marriage**				
Monogamous	146 (93.59)	175 (87.50)	3.666	0.056
Polygamous	10 (6.41)	25 (12.50)		
**Have children**				
Yes	157(76.96)	199 (90.87)	15.323	0.001*
No	47 (23.04)	20 (9.13)		
**Number of children**				
1 – 3	119 (75.80)	112 (56.28)	14.669	0.001*
>3	38 (24.20)	87 (43.72)		
**Driving experience (years)**				
≤ 10	181 (88.73)	94 (42.92)	97.412	0.001*
> 10	23 (11.27)	125 (57.08)		
**Average daily income (naira)**				
≤ 3500	177 (86.76)	97 (44.29)	83.501	0.001*
> 3500	27 (13.24)	122 (55.71)		
**Hypertension**				
Yes	14 (6.86)	7 (3.20)	3.009	0.083
No	190 (93.14)	212 (96.80)		
**Diabetes**				
Yes	13 (6.37)	5 (2.28)	4.335	0.037*
No	191 (93.63)	214 (97.72)		
**Heart disease**				
Yes	5 (2.45)	3 (1.37)	0.665	0.415
No	199 (97.55)	216 (98.63)		
**Perineal injury**				
Yes	193 (94.61)	201 (91.78)	1.322	0.250
No	11 (5.39)	18 (8.22)		
**Perineal surgery**				
Yes	1 (0.49)	4 (1.83)	1.615	0.204
No	203 (99.51)	215 (98.17)		
**Currently taking medication**				
Yes	16 (7.84)	9 (4.11)	2.648	0.104
No	188 (92.16)	210 (95.89)		
**Ever used alcohol**				
Yes	98 (48.04)	143 (65.30)	12.832	0.001*
No	106 (51.96)	76 (34.70)		
**Currently use alcohol**				
Yes	94 (46.08)	102 (46.58)	0.011	0.918
No	110 (53.92)	117 (53.42)		
**Ever smoked cigarette**				
Yes	24 (11.76)	45 (20.55)	5.969	0.015*
No	180 (88.24)	174 (79.45)		
**Ever used cannabis**				
Yes	24 (11.76)	19 (8.68)	1.104	0.293
No	180 (88.24)	200 (91.32)		
**Currently use cannabis**				
Yes	19 (9.31)	16 (7.31)	0.561	0.454
No	185 (90.69)	203 (92.69)		

* Level of significance at p < 0.05

The prevalence rates of erectile dysfunction among the long-distance commercial vehicle drivers and motorcycle riders were 47.4% and 71.4% respectively. The rate among CMRs was significantly higher than that in the long-distance drivers (χ2= 23.93, p = 0.001). Among motorcycle riders, correlates that were significantly associated with erectile dysfunction included marital status (χ2=11.56, p= 0.001), type of marriage (χ2= 4.30, p = 0.038), having children (χ2= 8.13, p = 0.004) and history of perineal injury (χ2= 6.99, p = 0.008) while age (χ2 = 5.19, p = 0.023), marital status (χ2= 6.57, p = 0.010), history of perineal injury (χ2= 7.46, p = 0.006) and current use of alcohol (χ2= 4.89, p = 0.027) were significantly associated with ED in long-distance drivers. Tables 2 & 3

**Table 2:** The association between sociodemographic/health-related characteristics and erectile dysfunction among commercial motorcycle riders

	ED Present (n=137)	ED Absent (n=55)	χ^2^	p-value
**Age (years)**				
< 40	102 (74.45)	37 (67.27)	1.012	0.314
≥ 40	35 (25.55)	18 (33.73)		
**Religion**				
Islam	63 (45.99)	29 (52.73)	0.715	0.398
Christianity	74 (54.01)	26 (47.27)		
**Educational status**				
Primary	36 (26.28)	13 (23.64)	1.277	0.528
Secondary	76 (55.47)	35 (63.64)		
Tertiary	25 (18.25)	7 (12.72)		
**Marital status**				
Not currently married	18(13.14)	19 (34.55)	11.559	0.001*
Currently married	119 (86.86)	36 (64.45)		
**Type of marriage**				
Monogamous	114 (95.80)	31 (86.11)	4.297	0.038*
Polygamous	5 (4.20)	5 (13.89)		
**Have children**				
Yes	117 (85.40)	37 (67.27)	8.125	0.004*
No	20 (14.60)	18 (32.73)		
**Number of children)**				
1 – 3	88 (75.21)	28 (75.68)	0.003	0.955
> 3	29 (24.79)	9 (24.32)		
**Driving experience** **(years)**				
≤ 10	123 (89.78)	46 (83.64)	1.405	0.236
> 10	14 (10.22)	9 (16.36)		
**Average daily income** **(naira)**				
≤ 3500	122 (89.05)	43 (78.18)	3.837	0.050
> 3500	15 (10.95)	12 (21.82)		
**Hypertension**				
Yes	12 (8.76)	2 (3.64)	1.524	0.217
No	125 (91.24)	53 (96.36)		
**Diabetes**				
Yes	10 (9.30)	3 (5.45)	0.212	0.646
No	127 (90.70)	52 (94.55)		
**Heart disease**				
Yes	4 (2.92)	1 (1.82)	0.188	0.665
No	133 (97.08)	54 (98.18)		
**Perineal injury**				
Yes	133 (97.08)	48 (87.27)	6.989	0.008*
No	4 (2.92)	7 (12.73))		
**Had perineal surgery**				
Yes	0 (0.00)	1 (1.82)	2.504	0.114
No	137 (100.00)	54 (98.18)		
**Currently taking medication**				
Yes	11 (8.03)	5 (9.09)	0.058	0.810
No	126 (91.97)	50 (90.91)		
**Ever used alcohol**				
Yes	73 (55.28)	23 (41.82)	2.064	0.151
No	64 (46.72)	32 (58.18)		
**Currently use alcohol**				
Yes	71 (51.82)	21 (38.18)	2.927	0.087
No	66 (48.18)	34 (61.82)		
**Ever smoked cigarette**				
Yes	14 (10.22)	9 (16.36)	1.405	0.236
No	123 (89.78)	46 (83.64)		
**Ever used cannabis**				
Yes	14 (10.22)	10 (18.18)	2.275	0.131
No	123 (89.78)	45 (81.82)		
**Currently use cannabis**				
Yes	11 (8.03)	8 (14.55)	1.869	0.172
No	126 (91.97)	47 (85.45)		

* Level of significance at p < 0.05

**Table 3:** The association between sociodemographic/health-related characteristics and erectile dysfunction among long-distance commercial vehicle drivers

	ED Present (n=102)	ED Absent (n=113)	χ^2^	P-value
**Age (years)**				
< 40	34 (33.33)	55 (48.67)	5.199	0.023*
≥ 40	68 (66.67)	58 (51.33)		
**Religion**				
Islam	53 (51.96)	70 (61.95)	2.184	0.139
Christianity	49 (48.04)	43 (38.05)		
**Educational status**				
Primary	32 (31.37)	42 (37.17)	1.085	0.581
Secondary	64 (62.75)	63 (55.75)		
Tertiary	6 (5.88)	8 (7.08)		
**Marital status**				
Not currently married	3 (2.94)	14 (12.39)	6.572	0.010*
Currently married	99 (97.06)	99 (87.612)		
**Type of marriage**				
Monogamous	89 (89.90)	84 (84.85)	1.145	0.285
Polygamous	10 (10.10)	15 (15.15)		
**Have children**				
Yes	97 (95.10)	99 (87.61)	3.731	0.053
No	5 (4.90)	14 (12.39)		
**Number of children**				
1 – 3	57 (58.76)	53 (53.54)	0.544	0.461
> 3	40 (41.24)	46 (46.46)		
**Driving experience** **(years)**				
≤ 10	40 (39.22)	50 (44.25)	0.558	0.455
> 10	62 (60.78)	63 (55.75)		
**Average daily income** **(naira)**				
≤ 3500	48 (47.06)	46 (40.71)	0.879	0.349
> 3500	54 (52.94)	67 (59.29)		
**Hypertension**				
Yes	4 (3.92)	2 (1.77)	0.915	0.339
No	98 (96.08)	111 (98.23)		
**Diabetes**				
Yes	3 (2.94)	2 (1.77)	0.324	0.569
No	99 (97.06)	111 (98.23)		
**Heart disease**				
Yes	3 (2.94)	0 (0.00)	3.371	0.066
No	99 (97.06)	113 (100.00)		
**Perineal injury**				
Yes	99 (97.06)	98 (49.75)	7.462	0.006*
No	3 (2.94)	15 (83.33)		
**Had perineal surgery**				
Yes	3 (2.94)	1 (0.89)	1.241	0.265
No	99 (97.06)	112 (99.11)		
**Currently taking medication**				
Yes	5 (4.90)	3 (2.66)	0.756	0.385
No	97 (95.10)	110 (97.34)		
**Ever used alcohol**				
Yes	65 (63.73)	77 (68.14)	0.466	0.495
No	37 (36.27)	36 (31.86)		
**Currently use alcohol**				
Yes	56 (54.90)	45 (39.82)	4.894	0.027*
No	46 (45.10)	68 (60.18)		
**Ever smoked cigarette**				
Yes	17 (16.67)	28 (24.78)	2.132	0.144
No	85 (83.33)	85 (75.22)		
**Ever used cannabis**				
Yes	8 (7.84)	11 (9.73)	0.238	0.626
No	94 (92.16)	102 (90.27)		
**Currently use cannabis**				
Yes	7 (6.86)	9 (7.96)	0.095	0.759
No	95 (93.14)	104 (92.04)		

* Level of significance at p < 0.05

Among motorcycle riders, binary logistic regression analysis showed that monogamous marriage type (OR = 4.69, p = 0.016) and history of perineal injury (OR = 4.73, p = 0.031) were significant predictors of ED whereas age 40 years and above (OR = 2.06, p = 0.024), history of perineal injury (OR = 7.73, p = 0.002) and current use of alcohol (OR = 2.48, p = 0.003) were significant of predictors of ED among long-distance vehicle drivers. Tables 4 & 5.

**Table 4:** Logistic regression analysis showing the sociodemographic and health-related predictors of erectile dysfunction among commercial motorcycle riders

Presence of Erectile Dysfunction	Odds Ratio (OR)	95% C.I	P value
**Marital status**			
Not currently married	Ref	0.84 – 32.84	0.077
Currently married	5.24		
**Type of marriage**			
Polygamous	Ref	0.06 – 0.75	0.016*
Monogamous	4.69		
**Have children**			
Yes	Ref	0.17 – 3.72	0.779
No	0.80		
**Perineal injury**			
No	Ref	1.15 – 19.39	0.031*
Yes	4.73		

* Level of significance at p < 0.05

**Table 5:** Logistic regression analysis showing the sociodemographic and health-related predictors of erectile dysfunction among long-distance commercial vehicle drivers.

Presence of Erectile Dysfunction	Odds Ratio (OR)	95% CI	P-value
**Age (years)**			
< 40	Ref	1.02 – 3.85	0.024*
≥ 40	2.06		
**Marital status**			
Not currently married	Ref	0.97 – 14.79	0.054
Currently married	3.80		
**Perineal injury**			
No	Ref	2.06 – 29.04	0.002*
Yes	7.73		
**Currently use alcohol**			
No	Ref	0.22 – 0.73	0.003*
Yes	2.48		

* Level of significance at p < 0.05

## Discussion

The prevalence of erectile dysfunction was significantly higher among commercial motorcycle riders compared with long-distance commercial vehicle drivers in Ibadan, Southwest Nigeria. While the prevalence among vehicle drivers was similar to that in the general population^2^,^5^, the prevalence among motorcycle riders was significantly higher. However, a study conducted in Japan among motorcyclists reported a prevalence similar to that of the commercial motorcycle riders in this study^13^. The significantly higher prevalence in the commercial motorcycle riders could be due to the effect of motorcycling on the riders which has been reported to cause a 2-fold increase in the risk of ED among motorcyclists^13^. Unlike the vehicle drivers, the compression of the perineum by the saddles and the vibration of the engines among motorcycle riders could damage the blood vessels and nerves, compromising the neurovascular supply to the penis and other perineal structures, thereby increasing the risk for ED^13^. Also, the design of the saddle has been reported to play a vital role in the pathophysiology of ED among motorcyclists^22^, but this was not explored in this study. Furthermore, the significantly lower average daily income among commercial motorcycle riders in this study, which is a measure of socioeconomic status could be a plausible reason for the higher prevalence among them as Seyam et al. had previously reported a significant association between erectile dysfunction and low socio-economic status among married Egyptian men^9^.

The correlates of ED among commercial motorcycle riders included marital status, marriage type, having children and a history of perineal injury. However, only monogamous marriage type and a history of perineal injury significantly predicted ED. Commercial motorcycle riders who had one wife were about 5 times at risk of ED compared to those with more than one wife. In this study, motorcycle riders in monogamous marriage settings had a higher occurrence of hypertension and diabetes compared to those in polygamous settings which could have contributed to the suboptimal erectile function, although this association was not significant. Consequently, they might be less motivated and adventurous in their sexual experiences with less engagement in sexual relationships, intercourse and multiple marital commitments. Commercial motorcycle riders with a self-reported history of blunt perineal injury associated with pain were about 5 times at risk of ED compared to those without a history of perineal injury, which is similar to the finding in the general population^5^.

Regarding long-distance commercial vehicle drivers, the correlates of ED were age, marital status, history of perineal injury and current use of alcohol, but only age, history of perineal injury and current use of alcohol significantly predicted ED. Increasing age predicted ED among long-distance vehicle drivers but not among motorcycle riders, with a two-fold increased risk among those aged 40 years and above. This could be attributed to the significantly older population of long-distance vehicle drivers compared to motorcycle riders who might be more at risk of comorbid medical conditions. Furthermore, the long-distance drivers who were currently using alcohol were about 3 times more likely to have ED compared to those who were currently not using alcohol. This was not surprising as the use of alcohol has been reported to significantly increase the risk for ED in the general population^5^,^6^,^12^. Long-distance commercial vehicle drivers with a history of blunt perineal injury associated with pain were almost 8 times at risk of ED compared to those without, which is similar to the finding in the general population^5^. There are some limitations to this study. Firstly, the cross-sectional design of this study makes the inference of causality between erectile dysfunction and the risk factors explored difficult. Secondly, the information obtained from the participants may have been biased by the inability to recall in detail some health-related events which could have been influenced by the transitory nature of their work.

Erectile dysfunction is more prevalent in the commercial motorcycle riders than the long-distance commercial vehicle riders. A self-reported history of blunt perineal injury or trauma associated with pain significantly predicted the development of ED in both groups. While increasing age and current use of alcohol were risk factors for ED in long-distance commercial vehicle drivers, monogamous marriage type was a risk factor for ED in commercial motorcycle riders. Therefore, efforts should be made to increase the awareness of ED among these populations to identify those at risk and institute the appropriate biological and psychosocial interventions. Furthermore, owing to the dearth of data on ED among motorcycle riders and vehicle drivers, more studies particularly prospective studies will be needed to provide more robust evidence that will possibly aid the inference of causality between ED and the associated factors with the view of developing health education tools on prevention strategies for erectile dysfunction.
